# Association between chemotherapy and the risk of developing breast cancer-related lymphedema: a nationwide retrospective cohort study

**DOI:** 10.1007/s00520-025-09169-3

**Published:** 2025-02-03

**Authors:** Sung Hoon Jeong, Seong Min Chun, Hyunji Lee, Miji Kim, Mira Choi, Ja-Ho Leigh

**Affiliations:** 1 Department of Rehabilitation Medicine, Seoul National University Hospital, 101 Daehak-Ro, Jongno-Gu, 03080 Seoul, Republic of Korea; 2 National Traffic Injury Rehabilitation Research Institute, National Traffic Injury Rehabilitation Hospital, Yangpyeong, 12564 Gyeonggi-Do Republic of Korea; 3 Institute of Health Services Research, Yonsei University, 50 Yonsei-Ro, Seodaemun-Gu, 03722 Seoul, Republic of Korea; 4 Department of Physical Medicine and Rehabilitation, Soonchunhyang University Hospital Seoul, Soonchunhyang University College of Medicine, 59 Daesagwan-Ro, 04401 Seoul, Republic of Korea; 5 Department of Rehabilitation Medicine, National Traffic Injury Rehabilitation Hospital, Yangpyeong, 12564 Gyeonggi-Do Republic of Korea; 6 Department of Biostatistics and Computing, Yonsei University Graduate School, 50 Yonsei-Ro, Seodaemun-Gu, 03722 Seoul, Republic of Korea; 7 Department of Dermatology, Ilsan Paik Hospital, Inje University College of Medicine, Goyang, 10380 Gyeonggi-Do Republic of Korea; 8 Institute of Health Policy and Management, Medical Research Center, Seoul National University, Seoul, Korea

**Keywords:** Breast cancer, Chemotherapy, Breast cancer-related lymphedema, Epidemiology

## Abstract

**Purpose:**

Breast cancer-related lymphedema (BCRL) is a well-known complication of breast cancer treatment, which often includes chemotherapy. This study aimed to investigate the association between chemotherapy and the risk of developing BCRL in patients with new-onset breast cancer.

**Methods:**

This nationwide retrospective cohort study utilized data from the Korean National Health Insurance database and the Korea National Cancer Incidence Database (2006–2017). Using 1:1 propensity score matching, 37,202 participants who received chemotherapy and 37,202 who did not receive chemotherapy were included in the analysis. Cox proportional hazard regression models were employed to examine the association between chemotherapy and the risk of developing BCRL.

**Results:**

Among the 74,404 participants, 11,508 (15.5%) were diagnosed with BCRL during the follow-up period. Compared with patients who did not receive chemotherapy, the risk of BCRL was higher in patients undergoing chemotherapy (hazard ratio [95% confidence interval]: 1.95 [1.87–2.04]). Furthermore, compared to patients who did not receive chemotherapy, the risk of BCRL was confirmed in the taxane (3.38 [3.19–3.58]), antimetabolite (1.79 [1.67–1.91]), and anthracycline (1.49 [1.41–1.56]) chemotherapy groups.

**Conclusion:**

Chemotherapy administration following a diagnosis of breast cancer increases the risk of BCRL. Therefore, vigilant monitoring for BCRL, particularly in patients undergoing chemotherapy with taxanes, antimetabolites, or anthracyclines, is warranted during follow-up.

**Supplementary Information:**

The online version contains supplementary material available at 10.1007/s00520-025-09169-3.

## Introduction

Breast cancer mortality has declined in recent decades because of improved screening and advancements in multidisciplinary treatment, such as surgery, radiotherapy, chemotherapy, and hormonal therapy [1]. Therefore, preventing complications to maintain and manage the quality of life of survivors has become a critical objective [2].

Breast cancer-related lymphedema (BCRL), a common complication following breast cancer treatment, is characterized by protein-rich fluid accumulation in the interstitial space of the ipsilateral upper limb [3]. Swelling and progressive fibrous skin changes can be debilitating and lead to a poor quality of life [3]. Factors related to BCRL occurrence include breast cancer-related procedures such as axillary lymph node dissection and sentinel lymph node surgery [4], chemotherapy, endocrine therapy [5], and radiotherapy targeting regional lymphatic vessels and nodes [6]. Modifiable lifestyle factors for BCRL include increased body mass index (BMI) and sedentariness [5]. Chemotherapy is often recommended for breast cancer. Neoadjuvant chemotherapy is increasingly administered before breast and axillary surgery, particularly in patients with high-risk tumor biology or positive nodules at diagnosis [3].

However, the association between chemotherapy utilization and the risk of developing BCRL has received limited attention [7]. Studies examining this association have often lacked comprehensive analysis, with conflicting results reported [1, 2, 7–9]. Previous studies found that specific chemotherapy agents, particularly taxanes such as docetaxel, can lead to fluid retention through mechanisms such as capillary protein leakage, potentially increasing the risk of lymphedema [8]. However, not all studies have found a significant association between chemotherapy and BCRL. A prospective cohort study concluded that taxane-based chemotherapy was not significantly associated with an increased risk of lymphedema compared to non-taxane regimens [2]. Moreover, previous studies have generally focused on specific chemotherapy regimens or were restricted to single or dual-center designs [1, 2, 8, 9]. To address these limitations, this study used a nationwide retrospective cohort to investigate the association between chemotherapy and the risk of developing BCRL in patients with new-onset breast cancer, without focusing on a specific regimen.

## Methods

### Data and study participants

This nationwide, population-based cohort study included all adult patients diagnosed with breast cancer in South Korea between 2006 and 2017, based on data from the National Health Insurance Service (NHIS) and the Korea National Cancer Incidence Database (KNCI DB). South Korea’s single-payer universal health coverage system ensures that > 99% of the population is covered by the NHIS, minimizing selection bias and providing comprehensive population-level data. The NHIS database includes detailed information on diagnoses, treatments, surgical history, prescriptions, demographics, socioeconomic status, and geographic data [10]. Previous studies have reported that the NHIS data demonstrate a sensitivity of 98.1% for breast cancer, confirming the high accuracy and completeness of the data [11]. The KNCI DB, maintained by the Korea Central Cancer Registry (KCCR), records all cancer diagnoses, including staging information, further enhancing the robustness of the data [12]. We linked the NHIS data with the KNCI DB records to enhance the information on primary cancer cases, a method that has been previously validated in studies for its reliability and completeness [13].

Data from 161,180 adult patients with breast cancer were gathered from historical cohorts within the NHIS and KNCI DB from January 1, 2006, to December 31, 2017. All patients were identified using the International Statistical Classification of Diseases and Related Health Problems, Tenth Revision (ICD-10) code C50 for breast cancer. A 1-year washout period (2006) was applied to identify patients with new-onset breast cancer. To minimize heterogeneity in clinical status, only patients undergoing breast cancer treatment (surgery, chemotherapy, or radiotherapy) within 6 months of the initial diagnosis were included. Patients diagnosed with lymphedema before or on the date of breast cancer treatment were excluded. Male patients and female patients aged < 20 years, and those with missing data on independent variables were also excluded. Finally, 126,528 individuals were identified, 89,326 of whom received chemotherapy within 6 months following the diagnosis of new-onset breast cancer; the remaining 37,202 participants did not receive chemotherapy.

**Fig. 1:**
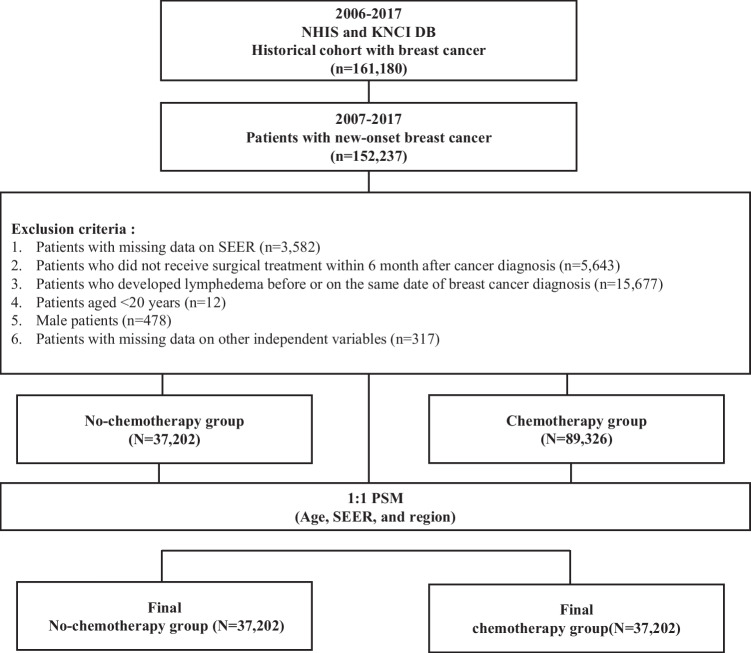
Study participant selection process. NHIS, National Health Insurance Service; KNCI DB, Korea National Cancer Incidence Database; SEER, Surveillance, Epidemiology, and End Results; PSM, propensity score matching

Propensity score matching (PSM) was employed to match participants who received chemotherapy within 6 months with those who did not. The propensity score used logistic regression to calculate the probability of receiving chemotherapy within 6 months, based on covariates of age; region; and Surveillance, Epidemiology, and End Results (SEER) stage. Following propensity score calculation, a 1:1 greedy match was established using the OneToManyMTCH macro for SAS (SAS Institute Inc., Cary, NC, USA) (Fig. 1) [14]. This study was exempted from ethics approval by the institutional review board of Seoul National University Hospital (IRB No. E-2206-076-1332) because the NHIS data were anonymized and de-identified. The requirement for participant informed consent was also waived.

### Study variables and covariates

The dependent variable was the occurrence of BCRL, defined by ICD-10 codes I972 and I890, identified at least once following a breast cancer diagnosis. The index date for each patient was the date of the first relevant treatment after breast cancer diagnosis. The follow-up period was from the index date to the date of lymphedema diagnosis, death, or the last follow-up (December 31, 2017), with an observation period of up to 5 years from the index date [5].

The primary variable of interest was the administration of chemotherapy within 6 months after breast cancer diagnosis [15]. Chemotherapy regimens included taxanes (docetaxel, paclitaxel), anthracyclines (doxorubicin, epirubicin), and antimetabolites (fluorouracil, capecitabine, cytarabine, methotrexate), and others (cyclophosphamide, vinorelbine, or carboplatin) (Online Resource 1). Participants were classified into a chemotherapy group if they received chemotherapy within 6 months of diagnosis and a no-chemotherapy group if they only received treatments other than chemotherapy (such as surgery and radiation).

Potential confounding factors included demographic, socioeconomic, health-related, and treatment-related variables influencing BCRL risk [16]. Demographic variables were age (≥ 50 or < 50 years, based on the menopausal age of Korean women) [17] and place of residence (urban, suburban, or rural) [18]. Urban areas included capital and metropolitan cities (≥ 500,000 residents), suburban areas included cities within provinces (50,000–500,000 residents), and rural areas had ≤ 50,000 residents. Socioeconomic factors were household income quartiles (low, mid-low, mid-high, high) and health insurance type (national health insurance [NHI] or medical aid). Approximately 97% of Koreans are NHI beneficiaries, while medical aid supports low-income or disabled individuals. Health-related factors included disability status, the Charlson Comorbidity Index (CCI), and SEER stage. Disabilities were categorized as Yes/No, based on Ministry of Health and Welfare standards [19]. The CCI, calculated using ICD-10 codes, grouped participants into scores of 0, 1, or ≥ 2 [20]. The SEER stage was classified as localized (code 1), regional (codes 2–4), distant (code 7), or unknown [21]. Treatment-related factors included the type of healthcare institution at the first visit (hospital, general hospital, tertiary hospital) and type of therapy received (surgical, radiation, hormonal, or targeted) (Online Resource 2).

### Statistical analysis

Considering the statistical properties of matched-pairs analysis, the association between chemotherapy and the risk of developing BCRL was analyzed in the final matched cohort. To assess covariate balance between the chemotherapy and no-chemotherapy groups, baseline characteristics were compared using standardized differences, with differences of < 0.1 (10%) generally considered negligible [22]. The frequency and percentage of each categorical variable for each patient were examined at baseline, and chi-square tests were performed to explore the distribution of BCRL incidence according to each variable. A Cox proportional hazards model was generated for examining the association between chemotherapy and the risk of developing BCRL. All Cox proportional hazards models were fully adjusted for the covariates presented in Table 1. Additionally, patients were categorized based on the type of chemotherapy received (taxane, anthracycline, antimetabolites, and others), and a Cox proportional hazards model was utilized to evaluate the risk of developing BCRL according to chemotherapy type (Online Resource 1). Analyses stratified by SEER, region, and household income level were conducted to investigate the association between chemotherapy and BCRL risk. Statistical significance was set at *P* < 0.05. All data were analyzed using SAS 9.4 (SAS Institute Inc; Cary, NC, USA).

**Table 1:** General characteristics of the study population according to the presence or absence of chemotherapy

Variables	Total		No-chemotherapy group		Chemotherapy group		Standardized difference
Total	N	%	N	%	N	%	
	74,404	(100.0)	37,202	(50.0)	37,202	(50.0)	
Chemotherapy							
No	37,202	(50.0)					
Yes	37,202	(50.0)					
Age (y)							0
≥ 50	30,616	(41.1)	15,308	(50.0)	15,308	(50.0)	
< 50	43,788	(58.9)	21,894	(50.0)	21,894	(50.0)	
Region							0
Urban	37,300	(50.1)	18,650	(50.0)	18,650	(50.0)	
Suburban	17,922	(24.1)	8,961	(50.0)	8,961	(50.0)	
Rural	19,182	(25.8)	9,591	(50.0)	9,591	(50.0)	
SEER							0
Localized	63,614	(85.5)	31,807	(50.0)	31,807	(50.0)	
Regional	8,210	(11.0)	4,105	(50.0)	4,105	(50.0)	
Distant	1,122	(1.5)	561	(50.0)	561	(50.0)	
Unknown	1,458	(2.0)	729	(50.0)	729	(50.0)	
Household income level							0.1521
Low	17,367	(23.3)	8,434	(48.6)	8,933	(51.4)	
Mid-low	12,997	(17.5)	5,865	(45.1)	7,132	(54.9)	
Mid-high	16,612	(22.3)	7,833	(47.2)	8,779	(52.8)	
High	27,428	(36.9)	15,070	(54.9)	12,358	(45.1)	
Health insurance							−0.0509
Medical aid	2,471	(3.3)	1,405	(56.9)	1,066	(43.1)	
NHI	71,933	(96.7)	35,797	(49.8)	36,136	(50.2)	
Disability							−0.079
No	70,451	(94.7)	34,896	(49.5)	35,555	(50.5)	
Yes	3,953	(5.3)	2,306	(58.3)	1,647	(41.7)	
Hospital level							0.1169
Hospital	36,186	(48.6)	19,077	(52.7)	17,109	(47.3)	
General hospital	21,910	(29.4)	10,138	(46.3)	11,772	(53.7)	
Tertiary hospital	16,308	(21.9)	7,987	(49.0)	8,321	(51.0)	
CCI							0.1083
0	20,888	(28.1)	9,685	(46.4)	11,203	(53.6)	
1	20,470	(27.5)	9,990	(48.8)	10,480	(51.2)	
≥ 2	33,046	(44.4)	17,527	(53.0)	15,519	(47.0)	
Surgery							−0.1398
No	3,693	(5.0)	1,283	(34.7)	2,410	(65.3)	
Yes	70,711	(95.0)	35,919	(50.8)	34,792	(49.2)	
Radiotherapy							−0.3486
No	33,283	(44.7)	13,465	(40.5)	19,818	(59.5)	
Yes	41,121	(55.3)	23,737	(57.7)	17,384	(42.3)	
Hormone therapy							−0.6754
No	62,655	(84.2)	26,987	(43.1)	35,668	(56.9)	
Yes	11,749	(15.8)	10,215	(86.9)	1,534	(13.1)	
Targeted therapy							0.4512
No	69,885	(93.9)	36,898	(52.8)	32,987	(47.2)	
YES	4,519	(6.1)	304	(6.7)	4,215	(93.3)	

*BCRL*, Breast cancer-related lymphedema; *SEER*, Surveillance, Epidemiology, and End Results; *NHI*, National Health Insurance; *CCI*, Charlson Comorbidity Index

Values are presented as number (%)

## Results

Table 1 presents the general characteristics of the participants after 1:1 PSM. The analysis included 74,404 participants, with 37,202 in the chemotherapy group and 37,202 in the no-chemotherapy group. Of these, 11,508 (15.5%) were diagnosed with BCRL during follow-up (Online Resource 3). The chi-square test indicated a significant difference in BCRL incidence between the groups. Factors such as age, region, SEER stage, household income level, healthcare institution type, CCI score, surgery, radiotherapy, hormone therapy, and targeted therapy were significantly associated with BCRL risk. However, no significant association was observed between the type of health insurance and the occurrence of disability-related BCRL (Online Resource 3).

Table 2 shows the results of the Cox proportional hazards regression models, which examined the association between chemotherapy and BCRL development risk after adjusting for the covariates listed in Table 1. After a breast cancer diagnosis, the BCRL risk was significantly higher in the chemotherapy group than in the no-chemotherapy group (hazard ratio [HR]: 1.95; 95% confidence interval [CI]: 1.87–2.04).

**Table 2:** Cox proportional hazards regression analysis of the association between chemotherapy and risk of developing BCRL in patients with breast cancer

Variables	Risk of developing BCRL			
	HR^*^	95% CI		
Chemotherapy				
No	1.00			
Yes	1.95	(1.87	–	2.04)
Age (y)				
≥ 50	1.00			
< 50	0.97	(0.94	–	1.01)
Region				
Urban	1.01	(0.97	–	1.06)
Suburban	1.14	(1.08	–	1.20)
Rural	1.00			
SEER				
Localized	1.00			
Regional	2.32	(2.21	–	2.43)
Distant	1.67	(1.47	–	1.89)
Unknown	1.62	(1.45	–	1.81)
Household income level				
Low	1.00			
Mid-low	1.02	(0.96	–	1.08)
Mid-high	0.94	(0.89	–	1.00)
High	0.92	(0.88	–	0.97)
Health insurance				
Medical aid	1.00			
NHI	1.08	(0.97	–	1.20)
Disability				
No	1.00			
Yes	1.02	(0.94	–	1.11)
Healthcare institution type				
Hospital	0.94	(0.90	–	0.99)
General hospital	1.02	(0.97	–	1.08)
Tertiary hospital	1.00			
CCI				
0	1.00			
1	1.02	(0.97	–	1.07)
≥ 2	1.12	(1.07	–	1.18)
Surgery				
No	1.00			
Yes	0.79	(0.74	–	0.86)
Radiotherapy				
No	1.00			
Yes	1.01	(0.97	–	1.05)
Hormone therapy				
No	1.00			
Yes	1.16	(1.09	–	1.23)
Targeted therapy				
No	1.00			
Yes	1.11	(1.03	–	1.18)

*BCRL*, Breast cancer-related lymphedema; *HR*, hazard ratio; *CI*, confidence interval; *SEER*, Surveillance, Epidemiology, and End Results; *NHI*, National Health Insurance; *CCI*, Charlson Comorbidity Index

^*^Adjusted for other covariates

**Fig. 2:**
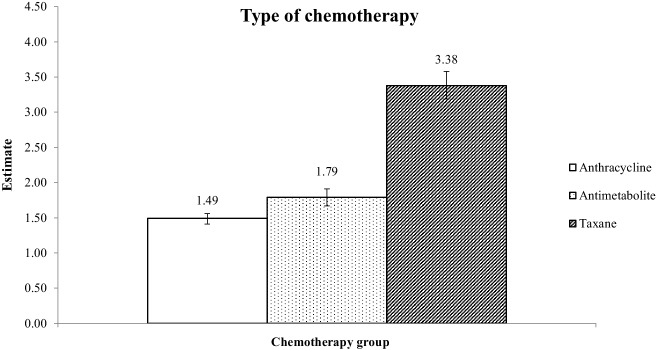
Association between the type of chemotherapy and the risk of developing BCRL in patients with breast cancer. BCRL, breast cancer-related lymphedema. ^*^Taxane group: Individuals receiving only taxane-based chemotherapy or chemotherapy, including taxane. **Anthracycline group:** Individuals receiving anthracycline-based chemotherapy, either exclusively or in combination with other agents, excluding taxane. **Antimetabolite group:** Individuals receiving antimetabolite-based chemotherapy, either exclusively or in combination with other agents, excluding taxane and anthracycline. **Other groups:** Individuals receiving chemotherapy that did not fall into the taxane, anthracycline, or antimetabolite groups; however, the group was not separately listed as no cases of lymphedema were observed

Figure 2 depicts the results of the subgroup analysis, illustrating the BCRL risk based on the chemotherapy administered during the study period. Compared to the no-chemotherapy group, the taxane therapy group (HR: 3.38; CI: 3.19–3.58), followed by the antimetabolite (HR: 1.79; CI: 1.67–1.91) and anthracycline (HR: 1.49; CI: 1.67–1.91) therapy groups, showed the highest risk of developing BCRL.

Table 3 presents the subgroup analysis examining the relationship between chemotherapy and BCRL risk stratified by SEER stage, region, and household income level. The association between chemotherapy and BCRL risk was the highest for patients with a low household income (HR [95% CI]: low, 2.09 [1.90–2.29]; mid-low, 2.07 [1.90–2.26]; mid-high: 1.86 [1.74–2.00]; high, 1.84 [1.66–2.03]), those with distant disease (HR [95% CI]: localized, 1.89 [1.80–1.99]; regional, 2.15 [1.95–2.37]; distant, 3.38 [2.57–4.45]; unknown, 2.24 [1.69–2.97]), and those residing in rural areas (urban: 1.79 [1.64–1.94]; suburban: 1.95 [1.84–2.08]; rural: 2.14 [1.96–2.33]).

**Table 3:** Subgroup analysis of the effect of chemotherapy on the risk of developing BCRL, stratified by SEER, region, and household income level

Variables		Risk of developing BCRL			
	HR	HR^*^	95% CI		
SEER					
Localized	1.00	1.89	(1.80	–	1.99)
Regional	1.00	2.15	(1.95	–	2.37)
Distant	1.00	3.38	(2.57	–	4.45)
Unknown	1.00	2.24	(1.69	–	2.97)
Region					
Urban	1.00	1.79	(1.64	–	1.94)
Suburban	1.00	1.95	(1.84	–	2.08)
Rural	1.00	2.14	(1.96	–	2.33)
Household income level					
Low	1.00	2.09	(1.90	–	2.29)
Mid-low	1.00	2.07	(1.90	–	2.26)
Mid-high	1.00	1.86	(1.74	–	2.00)
High	1.00	1.84	(1.66	–	2.03)

*BCRL*, Breast cancer-related lymphedema; *HR*, hazard ratio; *CI*, confidence interval; *SEER*, Surveillance, Epidemiology, and End Results; *CCI*, Charlson comorbidity Index

^*^Adjusted for other covariates

## Discussion

BCRL is a potential side effect of breast cancer treatment, resulting in functional impairment and psychosocial adverse effects in approximately 3–5 million patients per year worldwide [23]. These population-based findings provide valuable insights into BCRL risk after chemotherapy in Korean patients with breast cancer. The study results revealed that approximately 15.5% of patients who received chemotherapy after breast cancer diagnosis developed BCRL. Furthermore, BCRL risk was 1.95 times higher in the chemotherapy group than in the no-chemotherapy group. Among the various chemotherapy regimens, taxane therapy was associated with the highest risk. Notably, the impact of chemotherapy on BCRL occurrence was particularly pronounced among patients with a distant stage, low income, and low CCI score.

The reported BCRL incidence in patients with breast cancer varies widely (5–65%), depending on follow-up duration, treatment modality, and diagnostic criteria [24]. A recent Korean study using NHIS data reported a BCRL incidence of 12.2%, which is consistent with our findings [23]. However, other Korean studies reported a wider range (10–59%), likely due to the influence of single- or dual-center designs and short follow-up periods of 2–3 years [7, 23, 25]. Variations in BCRL assessment methods and study designs further complicate direct comparisons. Nevertheless, our study provides valuable insights into BCRL incidence over an extended period, using data representative of all patients with breast cancer in Korea.

Our results indicate that chemotherapy following a breast cancer diagnosis significantly increases BCRL risk. Notably, taxane chemotherapy was associated with the highest risk of BCRL, although antimetabolite and anthracycline chemotherapies also increased the risk. Several studies have investigated the relationship between chemotherapy and the risk of developing BCRL in patients with breast cancer, although results are conflicting [2, 5, 25]. These discrepancies have also been observed in studies exploring the link between specific types of anticancer treatments (such as taxane and anthracycline) and the risk of BCRL [2, 26]. We speculate that these discrepancies may arise from the inclusion of various surgical methods or radiation therapies rather solely examining the independent effects of chemotherapy. Our findings, suggesting that chemotherapy increases the BCRL risk, are consistent with those of previous studies. Notably, the elevated risk associated with taxane chemotherapy may be explained by previously proposed mechanisms. Taxane exposure can induce endothelial inflammation, leading to abnormal capillary permeability [27]. A previous study showed the development of edema in individuals undergoing taxane treatment, as observed through capillaroscopy and capillary filtration tests using 99mTc-labeled albumin [8]. Other researchers concluded that taxanes result in progressive protein accumulation in the interstitial space due to the abnormal capillary permeability, thus augmenting the interstitial extracellular fluid compartment and often leading to fluid retention in the extremities and generalized edema [28]. A clinical trial comparing taxane chemotherapy with other chemotherapy agents found that patients receiving taxane chemotherapy experienced swelling more frequently than did those receiving anthracycline or vinorelbine chemotherapy [25]. This information aligns with our finding that taxane chemotherapy was associated with a higher risk of developing BCRL than was antimetabolite or anthracycline chemotherapy.

Another study found that patients undergoing anthracycline-based chemotherapy were 1.49 times more likely to develop BCRL compared to those who did not [16]. A possible explanation for this is that anthracycline-based chemotherapy is associated with fluid retention, potentially contributing to lymphedema [26]. There is considerable evidence linking anthracyclines, particularly doxorubicin, with lymphedema development [16, 27, 29]. In a large cohort study evaluating risk factors for lymphedema in 1,794 patients with breast cancer, anthracycline treatment was associated with a 13.5% incidence of lymphedema over 5 years [27]. Additionally, in a prospective cohort study following 631 patients with breast cancer for 5 years, surgery and anthracycline chemotherapy increased the risk of lymphedema by four to five times [16]. Furthermore, the incidence of lymphedema more than doubled in patients who received anthracycline chemotherapy and underwent axillary lymphadenectomy and more than tripled in those who received radiation therapy [27]. Notably, doxorubicin binds to DNA double-helix base pairs, causing structural problems and preventing DNA degradation by topoisomerase-II. Furthermore, it generates hydroxyl free radicals, which have a cytotoxic effect, damaging lymph circulation and leading to lymphedema [30, 31]. Recent evidence has confirmed that doxorubicin impairs lymphatic flow by inhibiting the rhythmic contraction of lymphatic vessels [32]. This inhibition, along with lymphocyte retention, may damage lymphatic vascular structures, contributing to the long-term development of lymphedema [33, 34].

Our findings regarding antimetabolite-based chemotherapy increasing the risk of BCRL are also consistent with those previously reported. Antimetabolite drugs primarily inhibit DNA and RNA activity and may not directly contribute to the risk of developing BCRL. However, they can lead to complications such as infection and skin lesions, exacerbating impaired lymph node function, thereby increasing the BCRL risk. Studies have reported a higher risk of infection among women receiving a combination of 5-fluorouracil, methotrexate, and cyclophosphamide, as these drugs collectively reduce white blood cell count and lower the immune response [35]. Moreover, methotrexate inhibits DNA and RNA activity and interferes with cell division [36]. Therefore, the increased risk of developing BCRL in patients receiving these chemotherapy agents may be partially attributed to these conditions [37]. Further comprehensive studies are warranted to explore this indirect effect of antimetabolite-based chemotherapy on the BCRL risk.

Subgroup analysis showed that the detrimental effect of chemotherapy was particularly pronounced among patients from low-income households, those residing in rural areas, and those with a SEER stage of distant disease. Low socioeconomic status, including household income and residential location, can influence patient access to care and the availability of medical resources. In Korea, the unequal distribution of medical facilities, cutting-edge treatment facilities, and healthcare professionals between urban and rural areas is a significant concern [38]. Metropolitan areas with well-equipped medical facilities and higher doctor-to-patient ratios may allow for better treatment and easier follow-up care, which could mitigate the risk of BCRL [38]. Furthermore, individuals from low-income groups may face limited time for diagnosis and treatment due to financial constraints, impacting their treatment options [39]. Research has shown that these patients often undergo axillary surgery, significantly influencing the risk of developing BCRL [3]. High BMI, prevalent among rural residents in Korea, is a major factor in the occurrence of BCRL [40]. Furthermore, the higher risk of BCRL following chemotherapy in patients with advanced cancer stages, such as distant disease, may be attributed to the administration of a broader range and higher doses of medications in terminal patients [27, 41]. Patients with advanced terminal cancer may face a higher BCRL risk after receiving the same anticancer treatment due to challenges in preventive management resulting from pathological and comorbid conditions [42]. However, the role of chemotherapy in the relationship between cancer stage and BCRL warrants further research.

To the best of our knowledge, this study is one of the few to examine the relationship between cancer treatment and BCRL risk in Asian populations using national-level data. This large-scale cohort study, focused on the Korean population, overcomes the limitations of previous studies that were primarily single-center, restricted to specific cancer stages, or used national data with short follow-up periods [7, 23, 25]. By leveraging a comprehensive dataset representing the entire Korean breast cancer population and incorporating a longer follow-up, this study provides broader insights into chemotherapy-related complications [9, 23]. Notably, the use of PSM minimized confounding effects, enabling a more reliable assessment of the association between chemotherapy and BCRL [14]. Furthermore, unlike prior studies that focused on specific chemotherapy regimens, such as taxane or anthracycline, this research evaluated the effects of chemotherapy across various regimens [9, 26]. This study offers a comprehensive approach that addresses the limitations of earlier research by incorporating large-scale data, employing robust methods, and evaluating a range of chemotherapy regimens.

This study has some limitations. First, the disease code used as a selection criterion might not have accurately corresponded to the actual disease status of the patient, which is an inherent limitation of insurance databases [43]. However, almost all hospitals in Korea operate under a reimbursement system, and all surgical procedures and treatments are recorded in the claims database. To enhance diagnostic accuracy, both primary and secondary diagnostic codes were included in our analysis. Moreover, our study attempted to increase the accuracy of the diagnosis by connecting national-level data (NHIS and KNCI DB) [13]. Similar previous studies have shown that the sensitivity of NHIS data for breast cancer is high at 98.1% [44]. Second, the clinical information used in this study was limited as it relied on the available claims data. As a result, key variables such as BMI, physical activity, and the number of lymph nodes excised, which are significant contributors to BCRL risk, were not included, limiting the ability to control for important confounding factors [41]. However, this study comprehensively considered disability status, comorbidities, treatment types related to breast cancer, and SEER stage, which were often overlooked in previous studies [23]. Additionally, while the omission of these variables could potentially affect the robustness of the study results, we made every effort to mitigate these limitations by utilizing comprehensive, nationwide data [45]. Moreover, because of the inherent limitations of claims data, variables related to the availability of medical resources, healthcare access, and health surveillance systems—factors that may explain disparities in BCRL risk among rural and low-income populations—were not included in our dataset. Future research should aim to integrate such data to provide a more comprehensive understanding of the elevated BCRL risk observed in these populations. Third, the inability to include asymptomatic patients with BCRL or those who did not receive medical services might have introduced selection and unmeasured biases. To mitigate this bias and increase sample homogeneity, only patients with new-onset breast cancer were included using PSM. Fourth, the goal of this study was to examine the effect of chemotherapy alone. Therefore, the treatment sequence of chemotherapy before or after surgery, widely considered in previous studies, was not considered [3, 7, 16]. Nevertheless, the independent effect of chemotherapy on the risk of developing BCRL was examined by adjusting for surgery, radiation, hormone, and targeted therapy as covariates. Fifth, due to the retrospective nature of the data, causality could not be established. The observed increased risk of developing BCRL based on chemotherapy status may represent a temporal rather than causal relationship, necessitating caution when interpreting our findings. Finally, this study was conducted using data from Korea's single-payer healthcare system, which provides near-universal coverage. However, the findings may not be directly generalizable to regions with different healthcare systems or resource distributions. Racial and ethnic differences in lymphatic function or chemotherapy responses, which were not considered in this study, may also limit the generalizability of these results. Future studies in diverse populations and healthcare settings are needed to validate and expand upon these findings.

## Conclusion

Our findings validated chemotherapy as an independent risk factor for developing BCRL in patients with new-onset breast cancer. Taxane, antimetabolite, and anthracycline chemotherapies were associated with an increased BCRL risk, particularly among patients from low-income households, those living in rural areas, and those with distant disease stages. These findings highlight the need for vigilant monitoring and tailored interventions for patients undergoing chemotherapy after a breast cancer diagnosis.

## Supplementary Information

Below is the link to the electronic supplementary material.

Supplementary file1 (DOCX 17.9 KB)

Supplementary file2 (DOCX 17.6 KB)

Supplementary file3 (DOCX 29.5 KB)

## Data Availability

The data generated/analyzed during this study are available from the National Health Insurance Service (NHIS), but restrictions apply to the availability of these data, which were used under license for the current study and are not publicly available (NHIS-2022-1-470). Data are, however, available from the authors upon reasonable request and with permission of the NHIS. The results do not necessarily represent the opinion of the National Health Insurance Corporation.
