# Supplementary material for: Association between chemotherapy and the risk of developing breast cancer-related lymphedema: a nationwide retrospective cohort study
Source: Support Care Cancer. 2025 Feb 3;33(2):143. doi: 10.1007/s00520-025-09169-3 (PMC11790788; doi:10.1007/s00520-025-09169-3)
Supplement: Supplementary file 1 — Supplementary file1 (DOCX 17.9 KB) [file 520_2025_9169_MOESM1_ESM.docx]

***Supportive Care in Cancer***

**Association between chemotherapy and the risk of developing breast cancer-related lymphedema: A nationwide retrospective cohort study**

Sung Hoon Jeong, Seong Min Chun, Hyunji Lee, Miji Kim, Mira Choi, Ja-Ho Leigh

**Corresponding author**:

Ja-Ho Leigh, MD

Department of Rehabilitation Medicine, Seoul National University Hospital, 101 Daehak-ro, Jongno-gu, Seoul 03080, Republic of Korea

Tel: +82-31-580-5650

Fax: +82-2-2072-5244

Email: [jaho.leigh@gmail.com](mailto:jaho.leigh@gmail.com)

ORCID: <https://orcid.org/0000-0003-0465-6392>

**Online Resource 1. Electronic data interchange of chemotherapy regimens**

| **Type of chemotherapy** | **Definition** |
| --- | --- |
| **Taxane** | Korea drug codes:  docetaxel (148301BIJ, 148302BIJ, 148304BIJ, 148305BIJ, 148306BIJ),  148303BIJ, 148342BIJ, 148344BIJ, 148345BIJ, 148346BIJ, 148348BIJ, 148349BIJ, 148350BIJ, 148351BIJ, 148306BIJ, 148339BIJ);  paclitaxel (207801BIJ, 207802BIJ, 207803BIJ, 207804BIJ, 207805BIJ, 207806BIJ, 503701BIJ, 207831BIJ, 207832BIJ, 207830BIJ, 207833BIJ, 207834BIJ, 207835BIJ, 207836BIJ). |
| **Anthracycline** | Korea drug codes:  doxorubicin (149401BIJ, 149402BIJ, 149403BIJ, 149404BIJ, 149405BIJ, 149406BIJ, 149430BIJ, 149431BIJ, 149432BIJ, 149433BIJ, 149435BIJ, 149434BIJ);  epirubicin (152701BIJ, 152702BIJ, 152703BIJ, 152704BIJ). |
| **Antimetabolites** | Korea drug codes:  fluorouracil (161401BIJ, 161402BIJ, 161404BIJ, 161430BIJ, 161431BIJ, 161432BIJ);  capecitabine (122701ATB, 122702ATB);  gemcitabine (164901BIJ, 164902BIJ, 164903BIJ, 164930BIJ, 164931BIJ, 164932BIJ);  Cytarabine (139633BIJ, 139637BIJ, 139631BIJ, 139632BIJ, 139634BIJ, 139635BIJ, 139636BIJ, 139638BIJ, 139601BIJ, 139602BIJ);  methotrexate (192102BIJ, 192103BIJ, 192104BIJ, 192105BIJ, 192107BIJ, 192108BIJ, 192109BIJ, 192110BIJ, 192111BIJ, 192132BIJ, 192134BIJ, 192136BIJ, 192138BIJ, 192139BIJ, 192140BIJ, 192141BIJ, 192142BIJ, 192143BIJ, 192144BIJ). |
| **Others** | Korea drug codes:  cyclophosphamide (139004BIJ, 139005BIJ, 139001ATB, 139003BIJ);  mitomycin C (196401BIJ);  vinorelbine (248201BIJ, 248202BIJ);  Irinotecan (177430BIJ, 177431BIJ, 177433BIJ, 177435BIJ);  eribulin (621301BIJ);  carboplatin (123701BIJ, 123702BIJ, 123703BIJ, 123704BIJ, 123706BIJ, 123707BIJ, 123708BIJ);  cisplatin (134501BIJ, 134502BIJ, 134503BIJ, 134533BIJ, 134534BIJ). |
