# Supplementary material for: Association between chemotherapy and the risk of developing breast cancer-related lymphedema: a nationwide retrospective cohort study
Source: Support Care Cancer. 2025 Feb 3;33(2):143. doi: 10.1007/s00520-025-09169-3 (PMC11790788; doi:10.1007/s00520-025-09169-3)
Supplement: Supplementary file 2 — Supplementary file2 (DOCX 17.6 KB) [file 520_2025_9169_MOESM2_ESM.docx]

***Supportive Care in Cancer***

**Association between chemotherapy and the risk of developing breast cancer-related lymphedema: A nationwide retrospective cohort study**

Sung Hoon Jeong, Seong Min Chun, Hyunji Lee, Miji Kim, Mira Choi, Ja-Ho Leigh

**Corresponding author**:

Ja-Ho Leigh, MD

Department of Rehabilitation Medicine, Seoul National University Hospital, 101 Daehak-ro, Jongno-gu, Seoul 03080, Republic of Korea

Tel: +82-31-580-5650

Fax: +82-2-2072-5244

Email: [jaho.leigh@gmail.com](mailto:jaho.leigh@gmail.com)

ORCID: https://orcid.org/0000-0003-0465-6392

**Online Resource 2. Electronic data interchange of treatment modalities**

| **Variable** | **Definition** |
| --- | --- |
| **Surgery** | Korea health insurance classification codes (procedural codes):  N7130, N7131, N7132, N7133, N7134, N7135, P2121, P2122, P2123, P2124 |
| **Chemotherapy** | Korea drug codes: docetaxel (148301BIJ, 148302BIJ, 148304BIJ, 148305BIJ, 148306BIJ, 148303BIJ, 148342BIJ, 148344BIJ, 148345BIJ, 148346BIJ, 148348BIJ, 148349BIJ, 148350BIJ, 148351BIJ, 148306BIJ, 148339BIJ); Paclitaxel (207801BIJ, 207802BIJ, 207803BIJ, 207804BIJ, 207805BIJ, 207806BIJ, 503701BIJ, 207831BIJ, 207832BIJ, 207830BIJ, 207833BIJ, 207834BIJ, 207835BIJ, 207836BIJ); Doxorubicin (149401BIJ, 149402BIJ, 149403BIJ, 149404BIJ, 149405BIJ, 149406BIJ, 149430BIJ, 149431BIJ, 149432BIJ, 149433BIJ, 149435BIJ,149434BIJ); Epirubicin (152701BIJ, 152702BIJ, 152703BIJ, 152704BIJ), Vinorelbine (248201BIJ, 248202BIJ); Irinotecan (177430BIJ, 177431BIJ, 177433BIJ, 177435BIJ), Eribulin (621301BIJ); Capecitabine(122701ATB, 122702ATB); Carboplatin (123701BIJ, 123702BIJ, 123703BIJ, 123704BIJ, 123706BIJ, 123707BIJ, 123708BIJ); Cisplatin (134501BIJ, 134502BIJ, 134503BIJ, 134533BIJ, 134534BIJ); Cyclophosphamide (139004BIJ, 139005BIJ, 139001ATB,139003BIJ); Mitomycin C (196401BIJ); Cytarabine (139633BIJ, 139637BIJ, 139631BIJ, 139632BIJ, 139634BIJ, 139635BIJ, 139636BIJ, 139638BIJ, 139601BIJ, 139602BIJ); Gemcitabine (164901BIJ, 164902BIJ, 164903BIJ, 164930BIJ, 164931BIJ, 164932BIJ); Fluorouracil (161401BIJ, 161402BIJ, 161404BIJ,161430BIJ, 161431BIJ, 161432BIJ); Methotrexate(192102BIJ, 192103BIJ, 192104BIJ, 192105BIJ, 192107BIJ, 192108BIJ, 192109BIJ, 192110BIJ, 192111BIJ, 192132BIJ, 192134BIJ, 192136BIJ, 192138BIJ, 192139BIJ, 192140BIJ, 192141BIJ, 192142BIJ, 192143BIJ, 192144BIJ); |
| **Radiation therapy** | Korea health insurance classification codes (procedural codes): HD051, 052, 053. 054, 055, 056, 057, 058, 059, HD061, HZ271 |
| **Hormone therapy** | Korea drug codes: Tamoxifen (234501ATB, 234502ATB); Toremifene (242101ATB); Anastrozole (109001ATB); Letrozole (182201ATB); Exemestane (358401ATB); Leuprolide (182602BIJ, 182604BIJ); Goserelin (167201BIJ, 167202BIJ) |
| **Targeted therapy** | Korea drug codes: Abemaciclib (686603ATB); Bevacizumab (554330BIJ, 554331BIJ); Everolimus (485601ATB, 485602ATB, 485603ATB, 485604ATB); Lapatinib ditosylate (as lapatinib 0.25g) 507501ATB; palbociclib (655202ACH); Pembrolizumab (639001BIJ); pertuzumab (624601BIJ); Trastuzumab (242801BIJ, 242802BIJ, 242803BIJ, 626001BIJ, 626002BIJ). |
