# Supplementary material for: Association between chemotherapy and the risk of developing breast cancer-related lymphedema: a nationwide retrospective cohort study
Source: Support Care Cancer. 2025 Feb 3;33(2):143. doi: 10.1007/s00520-025-09169-3 (PMC11790788; doi:10.1007/s00520-025-09169-3)
Supplement: Supplementary file 3 — Supplementary file3 (DOCX 29.5 KB) [file 520_2025_9169_MOESM3_ESM.docx]

***Supportive Care in Cancer***

**Association between chemotherapy and the risk of developing breast cancer-related lymphedema: A nationwide retrospective cohort study**

Sung Hoon Jeong, Seong Min Chun, Hyunji Lee, Miji Kim, Mira Choi, Ja-Ho Leigh

**Corresponding author**:

Ja-Ho Leigh, MD

Department of Rehabilitation Medicine, Seoul National University Hospital, 101 Daehak-ro, Jongno-gu, Seoul 03080, Republic of Korea

Tel: +82-31-580-5650

Fax: +82-2-2072-5244

Email: [jaho.leigh@gmail.com](mailto:jaho.leigh@gmail.com)

ORCID: <https://orcid.org/0000-0003-0465-6392>

**Online Resource 3. General Characteristics of the Study Population Based on BCRL Risk**

| **Variables** | **Total** | | **Risk of BCRL** | | |  | **P-value** |
| --- | --- | --- | --- | --- | --- | --- | --- |
|  |  |  | **No** | | **Yes** | |  |
| **Total** | **74,404** | **(100.0)** | **62,896** | **(84.5)** | **11,508** | **(15.5)** |  |
| **Chemotherapy** |  |  |  |  |  |  | <.0001 |
| No | 37,202 | (44.4) | 33,174 | (89.2) | 4,028 | (10.8) |  |
| Yes | 37,202 | (44.4) | 29,722 | (79.9) | 7,480 | (20.1) |  |
| **Age** |  |  |  |  |  |  | <.0001 |
| ≥50 y | 30,616 | (36.5) | 25,932 | (84.7) | 4,684 | (15.3) |  |
| <50 y | 43,788 | (52.3) | 36,964 | (84.4) | 6,824 | (15.6) |  |
| **Region** |  |  |  |  |  |  | <.0001 |
| Urban | 37,300 | (44.5) | 31,676 | (84.9) | 5,624 | (15.1) |  |
| Sub-urban | 17,922 | (21.4) | 14,943 | (83.4) | 2,979 | (16.6) |  |
| Rural | 19,182 | (22.9) | 16,277 | (84.9) | 2,905 | (15.1) |  |
| **SEER** |  |  |  |  |  |  | <.0001 |
| Localized | 63,614 | (75.9) | 55,067 | (86.6) | 8,547 | (13.4) |  |
| Regional | 8,210 | (9.8) | 5,846 | (71.2) | 2,364 | (28.8) |  |
| Distinct | 1,122 | (1.3) | 844 | (75.2) | 278 | (24.8) |  |
| Unknown | 1,458 | (1.7) | 1,139 | (78.1) | 319 | (21.9) |  |
| **Household income level** |  |  |  |  |  |  | <.0001 |
| Low | 17,367 | (20.7) | 14,533 | (83.7) | 2,834 | (16.3) |  |
| Mid-low | 12,997 | (15.5) | 10,813 | (83.2) | 2,184 | (16.8) |  |
| Mid-high | 16,612 | (19.8) | 14,045 | (84.5) | 2,567 | (15.5) |  |
| High | 27,428 | (32.7) | 23,505 | (85.7) | 3,923 | (14.3) |  |
| **Health insurance** |  |  |  |  |  |  | 0.3709 |
| Medical aid | 2,471 | (2.9) | 2,073 | (83.9) | 398 | (16.1) |  |
| NHI | 71,933 | (85.8) | 60,823 | (84.6) | 11,110 | (15.4) |  |
| **Disability** |  |  |  |  |  |  | 0.4809 |
| No | 70,451 | (84.1) | 59,570 | (84.6) | 10,881 | (15.4) |  |
| Yes | 3,953 | (4.7) | 3,326 | (84.1) | 627 | (15.9) |  |
| **Healthcare institution type** | |  |  |  |  |  | <.0001 |
| Hospital | 36,186 | (43.2) | 30,915 | (85.4) | 5,271 | (14.6) |  |
| General hospital | 21,910 | (26.1) | 18,271 | (83.4) | 3,639 | (16.6) |  |
| Tertiary hospital | 16,308 | (19.5) | 13,710 | (84.1) | 2,598 | (15.9) |  |
| **CCI** |  |  |  |  |  |  | 0.0002 |
| 0 | 20,888 | (24.9) | 17,743 | (84.9) | 3,145 | (15.1) |  |
| 1 | 20,470 | (24.4) | 17,417 | (85.1) | 3,053 | (14.9) |  |
| ≥2 | 33,046 | (39.4) | 27,736 | (83.9) | 5,310 | (16.1) |  |
| **Surgery** |  |  |  |  |  |  | <.0001 |
| No | 3,693 | (4.4) | 2,818 | (76.3) | 875 | (23.7) |  |
| Yes | 70,711 | (84.4) | 60,078 | (85.0) | 10,633 | (15.0) |  |
| **Radiotherapy** |  |  |  |  |  |  | <.0001 |
| No | 33,283 | (39.7) | 27,425 | (82.4) | 5,858 | (17.6) |  |
| Yes | 41,121 | (49.1) | 35,471 | (86.3) | 5,650 | (13.7) |  |
| **Hormone therapy** |  |  |  |  |  |  | <.0001 |
| No | 62,655 | (74.8) | 52,681 | (84.1) | 9,974 | (15.9) |  |
| Yes | 11,749 | (14.0) | 10,215 | (86.9) | 1,534 | (13.1) |  |
| **Targeted therapy** |  |  |  |  |  |  | <.0001 |
| No | 69,885 | (83.4) | 59,307 | (84.9) | 10,578 | (15.1) |  |
| YES | 4,519 | (5.4) | 3,589 | (79.4) | 930 | (20.6) |  |

BCRL, Breast cancer-related lymphedema; NHI, National health insurance; CCI, Charlson Comorbidity Index.

Values are presented as number (%).
